# Prediction of Function Determining and Buried Residues Through Analysis of Saturation Mutagenesis Datasets

**DOI:** 10.3389/fmolb.2021.635425

**Published:** 2021-03-11

**Authors:** Munmun Bhasin, Raghavan Varadarajan

**Affiliations:** ^1^ Molecular Biophysics Unit, Indian Institute of Science, Bangalore, India; ^2^ Jawaharlal Nehru Centre for Advanced Scientific Research, Bangalore, India

**Keywords:** deep sequencing, saturation mutagenesis, protein function, activity, stability, phenotype

## Abstract

Mutational scanning can be used to probe effects of large numbers of point mutations on protein function. Positions affected by mutation are primarily at either buried or at exposed residues directly involved in function, hereafter designated as active-site residues. In the absence of prior structural information, it has not been easy to distinguish between these two categories of residues. We curated and analyzed a set of twelve published deep mutational scanning datasets. The analysis revealed differential patterns of mutational sensitivity and substitution preferences at buried and exposed positions. Prediction of buried-sites solely from the mutational sensitivity data was facilitated by incorporating predicted sequence-based accessibility values. For active-site residues we observed mean sensitivity, specificity and accuracy of 61, 90 and 88% respectively. For buried residues the corresponding figures were 59, 90 and 84% while for exposed non active-site residues these were 98, 44 and 82% respectively. We also identified positions which did not follow these general trends and might require further experimental re-validation. This analysis highlights the ability of deep mutational scans to provide important structural and functional insights, even in the absence of three-dimensional structures determined using conventional structure determination techniques, and also discuss some limitations of the methodology.

## Introduction

Mutagenesis is a tool to learn about proteins, identifying functionally significant protein positions, and understanding determinants of protein folding and stability. Deep mutational scanning involving a combination of saturation mutagenesis, phenotypic screening and next generation sequencing allows high-throughput analysis by measuring the effects of all possible amino acid substitutions on protein function (Fowler and Fields, 2014). Deep mutational scanning reveals the impact of mutations on a specific protein property, for example, interaction with a partner protein or enzymatic activity. A general workflow for a deep mutational scan involves the creation of a library of variants by applying a mutagenesis protocol to the genetic region of interest (Fowler et al., 2010) which can include an entire coding sequence (Adkar et al., 2012). Next, these libraries are subjected to some selection pressure, and this is used to observe the change in the frequency of variants with a particular phenotype. The libraries are sequenced before and after selection to obtain relative occurrences of different mutants in the population and estimate relative enrichment with respect to the wild type sequence (Tripathi and Varadarajan, 2014).

There have been numerous attempts to understand and predict functional consequences of mutations by using computational methods (Bloom et al., 2005; Moretti et al., 2013). The availability of deep mutational scanning data has helped to understand the contribution of every amino acid in a protein to its structure, stability, and function, understand how these mutations regulate protein activity, and to build on this information to predict functional effects of mutations in other contexts. Mutations can affect activity either by altering the specific activity, altering the level of properly folded protein *in vivo*, or by a combination of the above (Tripathi et al., 2016). Identifying which of these is the primary contributor to an observed phenotype is non-trivial.

For understanding the functional role of a protein, it is essential to identify the key catalytic or functionally important residues that we collectively refer to as active-site residues. There are several tools available to predict protein function based on query protein sequence or structural homology with well-characterized proteins (Gherardini and Helmer-Citterich, 2008). One of the common methods used to identify catalytic sites is using sequence conservation (Berezin et al., 2004; Fischer et al., 2008). With the availability of three-dimensional structures of proteins, these methods can be further improved by combining structural and sequence conservation information (Lichtarge et al., 1996; Aloy et al., 2001; Capra et al., 2009). These methods provide cues to design experiments, including site-directed mutagenesis experiments, and help to give an improved prediction of function (George et al., 2005). Such methods are helpful in cases where protein structural information is available. For cases with insufficient structural information, the data from deep mutational scans can be utilized in order to infer functional sites based on the substitution preferences across the protein under study.

In the present study, we have analyzed several deep mutational scanning datasets and observed the mutational sensitivity patterns at buried and exposed positions. Further, the sequence-based predicted accessibility values were incorporated together with the mutational sensitivity scores to predict functional or active-site residues. These residues include residues involved in catalytic activity, substrate binding, as well as protein-protein or protein-ligand interactions. Predicted accessibility scores help in the separation of the exposed from the buried residues. Residues that are sensitive to mutation and predicted to be exposed are likely to constitute the active-site, while the remaining mutationally sensitive residues are likely to be buried.

## Materials and Methods

### Datasets for Large-Scale Mutagenesis

A subset of the published deep mutational scanning datasets was curated. The result was a set of 12 deep mutational scans (Table 1). While several other studies have been published, most lack sufficient coverage of single-site mutations over the region of interest, have more than one mutation per read or describe complex phenotypes which preclude easy interpretation of the data. Alternatively, several studies report heatmaps and raw sequencing data without having the underlying numerical values of the processed enrichment scores publicly available.

**TABLE 1:** Large-scale deep mutational scanning datasets used in this study.

Data set	Mutagenized positions	Host	Selection	PDB ID	Citation
Aminoglycoside kinase	264	*E. coli.*	Antibiotic resistance	1ND4	(Melnikov et al. (2014))
BRCA1 RING domain-BARD1 binding	102	*S. cerevisiae*	Binding activity (Y2H)	1JM7	(Starita et al. (2015))
BRCA1 RING domain–E3 ligase activity	102	*S. cerevisiae*	Ubiquitin ligase activity	1JM7	(Starita et al. (2015))
CcdB	100	*E. coli.*	Toxin activity	3VUB	(Adkar et al. (2012))
Gal4 (DBD)	64	*S. cerevisiae*	Transcription factor activity	3COQ	(Kitzman et al. (2015))
G protein (GB1-IgG-Binding domain)	54	*Streptococcus sp. group G*	IgG-Fc binding	1PGA	(Olson et al., (2014))
Hsp90 (ATPase domain)	219	*S. cerevisiae*	Chaperone activity	2CG9	(Mishra et al. (2016))
NUDT15	163	*E. coli*	Abundance and drug sensitivity	5LPG	(Suiter et al. (2020))
Pab1 (RRM domain)	75	*S. cerevisiae*	mRNA binding	1CVJ	(Melamed et al. (2013))
PSD95(pdz3 domain)	83	*E. coli.*	Ligand binding	1BE9	(McLaughlin et al. (2012)
TEM1 β-lactamase	263	*E. coli*	Antibiotic resistance	1FQG	(Stiffler et al. (2015))
Ubiquitin	75	*S. cerevisiae*	Ubiquitin ligase activity	1UBQ	(Roscoe et al. (2013))

### Data Rescaling

Most of the deep mutational scanning datasets reported mutational effect scores as the log-transformed ratio of mutant frequency before and after selection, divided by wild-type frequency before and after selection. The counts/frequency of the mutational sensitivity scores were considered from the original datasets, and their distribution was plotted. The values were sorted, and the 5th percentile of the value was taken as the minimum value, min*(M)*, for rescaling. The maximum value, max*(M)*, for the rescaling was considered as the value at the peak for the wild type in the histograms. This peak arises because many mutational effect scores are close to that of the WT. The scores were rescaled between 0 and −1 using the formula:

$$\;M_{rescaled}=\left(b-a\right)\frac{M-\min\left(M\right)}{\max\left(M\right)-\min\left(M\right)}+a,$$

where, *M* is the mutational effect score, *a* and *b* are −1 and 0, respectively. With this normalization, the most sensitive positions have mutational effect score ≈−1 and the wild type like mutations have mutational effect score ≈0 (Supplementary Figure S1 and Supplementary Table S1).

### Depth and Accessibility Calculations

Both depth and accessibility of each residue were calculated from the available structures deposited in the Protein Data Bank. Amongst the datasets in the study, five of the proteins had high-resolution PDB structures, namely dimeric CcdB structure (PDB ID 3VUB) (Loris et al., 1999), PSD95 pdz3 domain (PDB ID 1BE9), BRCA1 RING domain (PDB ID 1JM7) (Starita et al., 2015), Gal4 (PDB ID 3COQ) (Marmorstein et al., 1992) and TEM1 β-lactamase (PDB ID 1FQG) (Strynadka et al., 1992).

The residue depth calculations were performed using the DEPTH server (http://cospi.iiserpune.ac.in/depth/htdocs/index.html) (Chakravarty and Varadarajan, 1999; Tan et al., 2011). A residue was defined as buried or exposed if the side chain accessibility is ≤5 or >5% respectively, based on the accessibility calculated using the NACCESS program (Adkar et al., 2012).

### Prediction of Sequence-Based Surface Accessibility

The sequence-based surface accessibility values were predicted using PROF (Rost and Sander, 1994), a neural network-based method (https://open.predictprotein.org/). These values were compared with the structure-based surface accessibility values, which were calculated using the NACCESS program (Hubbard and Thornton, 1993). NetSurfP was also used for the prediction of sequence-based surface accessibility (Petersen et al., 2009) and compared with the prediction results obtained using PROF. PROF and NetSurfP predictions were also compared with SPIDER3 (Heffernan et al., 2017), a machine learning method that takes into account the non-local interactions in its predictions.

### Prediction of the Active-Site, Buried and Exposed Non Active-Site Residues

The rescaled mutational sensitivity values were averaged across mutations for each position. The averaged mutational sensitivity scores were filtered to include only those positions that had mutational data for a minimum of 10 mutants per position. Also, only those positions were considered for which the predicted sequence-based accessibility values were predicted. Both the scores for averaged mutational sensitivity and PROF accessibility are converted to Z-scores by subtracting the mean value and dividing by the standard deviation. The final score for predicting the active-site residues is obtained by using the following formula:

$$\;Z_{\text{pred}}=Z_{{\text{average}}_{\text{mut}-\text{sens}}}\pm Z_{\text{prof}-\text{acc}},$$

Where, *Z* represents the z-scores. For the prediction of active-site residues, the two scores are added, whereas the scores are subtracted for the prediction of buried positions. The mean and standard deviation were calculated for the combined score. Residues with scores one standard deviation away from the mean were predicted as active-site or buried.

For the prediction of exposed non-active site residues, the same scores that used the rescaled averaged mutational sensitivity scores along with the sequence-based accessibility scores were considered. The residues that occurred beyond the cut-off for prediction of active-site residues were predicted to be exposed non active-site residues. A similar analysis was performed by incorporating the sequence-based accessibility values obtained using NetSurfP and SPIDER3 to compare the three classes of prediction namely, active-site, buried and exposed non active-site residues.

### Evaluation Metrics

We assume the active-site residues to represent the positive samples and non active-site residues to represent the negative samples for the prediction of active-site residues. On the other hand, for the prediction of the buried sites, we consider the buried site residues as the positive samples and the exposed residues as the negative samples. The exposed non active-site prediction considered the positive and negative samples in similar way. To evaluate the performance of prediction, four evaluation metrics are used in this study: sensitivity, specificity, accuracy, and Matthews correlation coefficient (MCC).

$$\mathrm{Sensitivity}=\frac{\mathrm{TP}}{\mathrm{TP}+\mathrm{FN}},$$

$$\mathrm{Specificity}=\frac{\mathrm{TN}}{\mathrm{TN}+\mathrm{FP}},$$

$$\mathrm{Accuracy}=\frac{\mathrm{TP}+\mathrm{TN}}{\mathrm{TP}+\mathrm{TN}+\mathrm{FP}+\mathrm{FN}},$$

$$\mathrm{Matthews Correlation Coefficient}=\frac{\left(\mathrm{TP}*\;\mathrm{TN}\right)-\left(\mathrm{FP}*\;\mathrm{FN}\right)}{\sqrt{\left(\mathrm{TP}+\mathrm{FP}\right)\left(\mathrm{TP}+\mathrm{FN}\right)\left(\mathrm{TN}+\mathrm{FP}\right)\left(\mathrm{TN}+\mathrm{FN}\right)}},$$

where TP, TN, FP, FN are True Positives, True Negatives, False Positives, and False Negatives, respectively.

## Results

**FIGURE 1:**
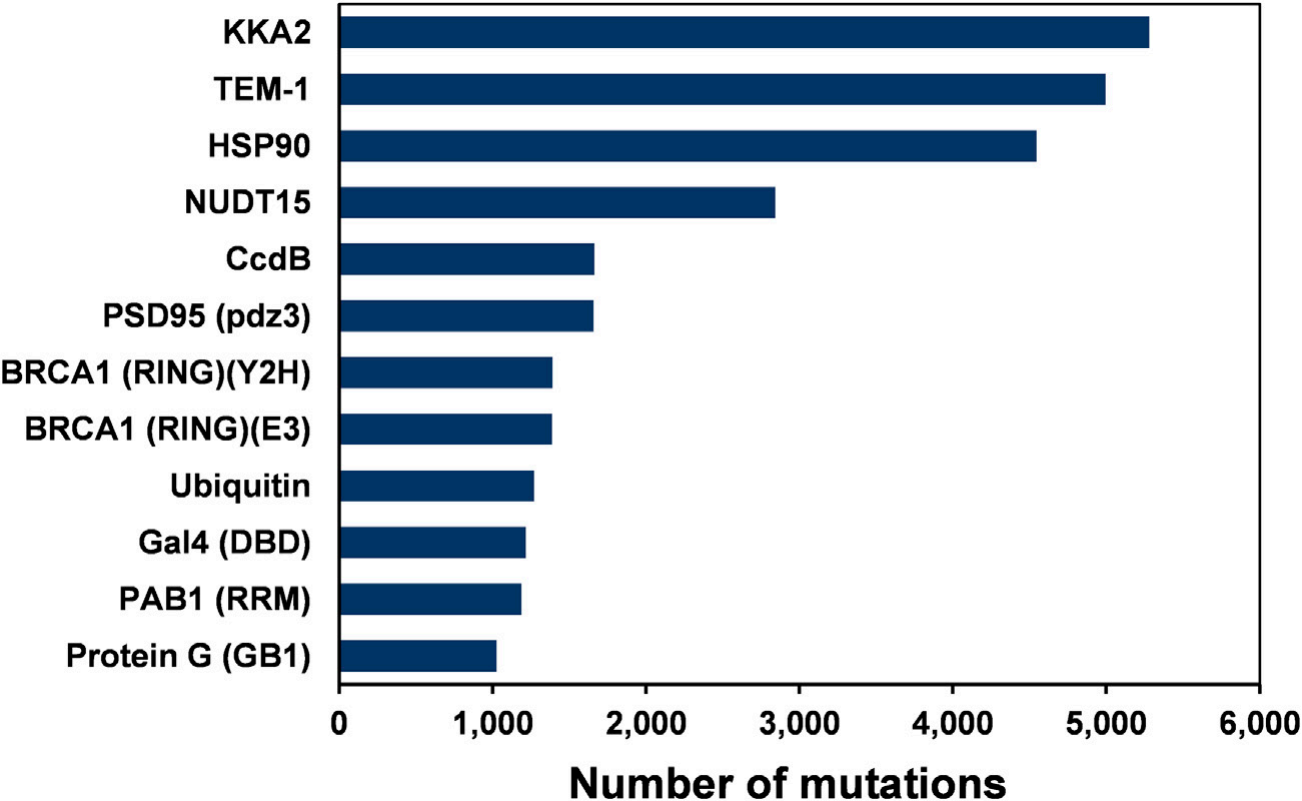
The number of single amino-acid mutations in various deep mutational scanning datasets of 12 proteins.

Deep mutational scanning involves measurement of large numbers of mutational phenotypes for a given protein using phenotypic screening coupled to deep sequencing (Adkar et al., 2012; Fowler et al., 2010). It can be used to quantify the phenotypic effects of all mutations at each position in a protein. These deep mutational scanning data sets help understand the relationships between amino acid sequence and phenotype. The assay formats used for the deep mutational scans included plate-based activity screens, FACS, phage display and yeast two-hybrid methodologies (Gupta and Vardarajan, 2018). Site-saturation mutagenesis (SSM) has been employed in several studies to probe residue-specific contributions to activity, stability, and binding for whole proteins (Gray et al., 2017). This study analyzed 12 large-scale mutational datasets of 11 proteins from existing deep mutational scan experiments (Figure 1 and Table 1). In the case of BRCA1, there are two independent deep mutational scan experiments, one for BRCA1 BARD1 binding and the other for E3 ligase activity (Starita et al., 2015). In these separate experiments, a multiplexed yeast two-hybrid assay was used to select for the ability of BRCA1 RING domain (2–103) variants to interact with the RING domain of BARD1. The structure is also available for the BRCA1/BARD1 RING-domain heterodimer (1JM7) (Brzovic et al., 2001). Since the present study involves the prediction of the active-site residues based on the mutational effect scores and the sequence-based accessibility predictions, the variants from the same region (2–103) of BRCA1 were used for the E3 ligase function experiment instead of using the E3 ligase scores available for full-length BRCA1 protein.

Some general patterns of mutational sensitivity were observed for the datasets used in the present study. Buried residues have high mutational sensitivity compared to those that are exposed and not part of the active-site. The residues that show high mutational sensitivity at exposed regions are typically involved in an interaction with some other proteins or are part of a catalytic or ligand binding site. As discussed above, these residues are classified as active-site residues. Hence, it is important to examine if these active-site residues can be distinguished from buried residues based on the mutational sensitivity scores, even in the absence of structural data (Tripathi et al., 2016).

### Analysis of Mutational Sensitivity Data

The datasets contained effect scores for most mutations at each position. To facilitate comparisons between each data set, the mutational effect scores were rescaled for each protein. To understand the overall trends in mutational sensitivity, the substitution preferences were examined for all the proteins in the dataset. A residue was defined as buried or exposed based on its side-chain accessibility calculated using the NACCESS program. A cut-off of 5% side-chain accessibility was used (Adkar et al., 2012). The interface residues for the proteins in the dataset were determined from the corresponding literature citations of their respective structures.

Most exposed positions have a low mutational sensitivity (Supplementary Figure S2). It has been observed that buried residues along with some of the exposed residues have a high mutational sensitivity. Exposed residues that are sensitive to mutations are likely to be a part of the active-site (Wu et al., 2015). We examined if the substitution specific patterns of mutational sensitivity could help to distinguish the active-site residues from the buried ones. The effect of various substitutions was analyzed for different categories, namely aliphatic, aromatic, polar and charged (Supplementary Figure S2). In most cases, buried positions tolerated aliphatic substitutions, except when the wild-type residue is an Alanine or Glycine residue. Polar and charged residues are poorly tolerated at buried positions. Exposed active-site residues showed very high mutational sensitivity including for substitutions to aliphatic residues (Supplementary Figure S2). The general trends in mutational sensitivity were similar for most proteins that were considered for the analysis. However, some proteins namely E3 ligase activity of BRCA1 RING domain, NUDT15 and aminoglycoside kinase were exceptionally sensitive to mutation, even at exposed non active-site residues. Even for the same protein, two different activity assays namely BARD1 binding and E3 ligase activity showed very different mutational sensitivity profiles. While this is understandable for active-site residues, it is hard to understand for buried residues where mutations are expected to primarily affect protein levels, rather than specific activity (Bajaj et al., 2008; Tripathi et al., 2016)

### Correlation Between Calculated and Predicted Solvent Accessibility

To predict the active-site residues solely from the mutational sensitivity data, the accessibility was predicted based on sequence using PROF (Rost and Sander et al., 1994). Further, the correlation was calculated between the calculated surface accessibility and the predicted accessibility values (Table 2). The predicted surface accessibility for the 11 proteins from 12 datasets showed a Pearson’s correlation coefficient r ∼ 0.6 with the calculated surface accessibility values in most cases. The predicted accessibility information was combined with the mutational sensitivity scores to predict the active-site and buried residues as described in the Methods section.

**TABLE 2:** Correlation coefficients of surface accessibility predicted using PROF, NetSurfP and SPIDER3 with values calculated from the structure using NACCESS. The oligomeric state of the protein based on the PDB structure is also mentioned.

Protein	Correlation coefficient			Oligomeric state
	PROF	NetSurfP	SPIDER3	
Aminoglycoside kinase	0.66	0.75	0.69	Dimer
BRCA1 RING domain	0.45	0.62	0.66	Monomer
CcdB	0.71	0.75	0.74	Dimer
Gal4 (DBD)	0.73	0.77	0.66	Tetramer
GB1 (IgG-binding domain)	0.67	0.52	0.64	Monomer
Hsp90 (ATPase domain)	0.56	0.64	0.59	Tetramer
NUDT15	0.55	0.63	0.62	Dimer
Pab1 (RRM domain)	0.75	0.81	0.77	Dimer
PSD (pdz3 domain)	0.74	0.81	0.61	Dimer
TEM1 β- lactamase	0.74	0.81	0.79	Monomer
Ubiquitin	0.74	0.84	0.73	Monomer

**FIGURE 2:**
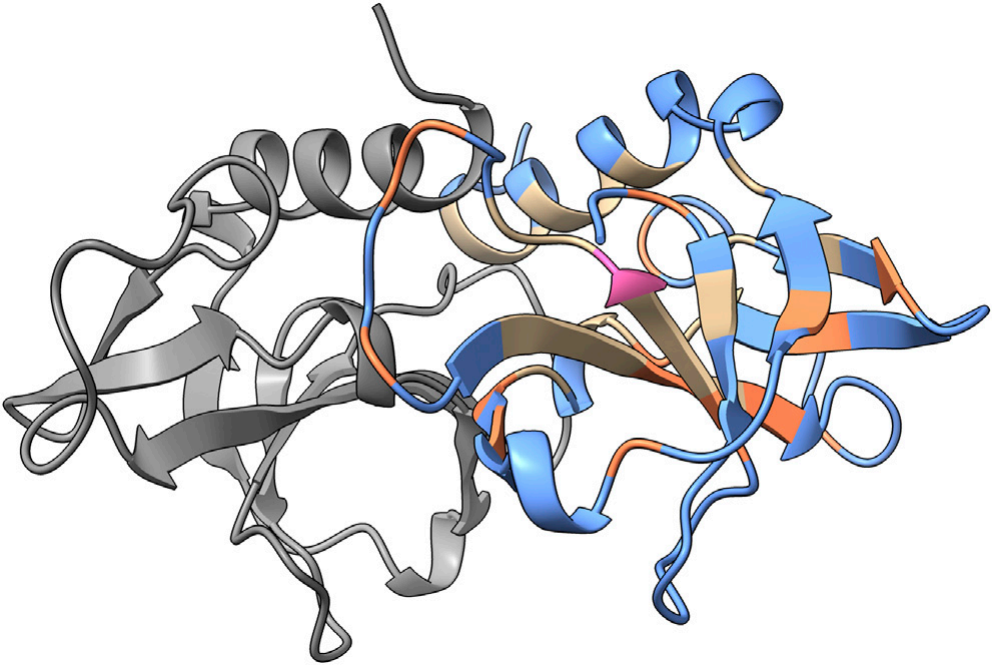
PROF prediction results for CcdB. The sequence-based surface accessibility results obtained from PROF mapped onto the structure of CcdB homodimer (PDB ID: 3VUB). The predictions with respect to the exposed positions are mapped on the structure. One monomer is highlighted in gray and the prediction results are mapped onto the other monomer. The true positives are highlighted in blue, false positives in pink, true negatives in tan and false negatives in orange based on the predictions from PROF and crystal structure accessibilities calculated using NACCESS.

To illustrate the accuracy of the accessibility predictions, results obtained from PROF and the calculated accessibility from NACCESS are mapped on the structure of CcdB (PDB ID: 3VUB). CcdB is a 101-residue homodimeric toxin found on F-plasmid (Figure 2). The true positives, false positives, true negatives and false negatives are highlighted in the figure. Here, true positives are correctly predicted exposed residues while false positives are buried residues that are incorrectly predicted as exposed by PROF. True negatives were correctly predicted buried residues and false negatives were exposed residues wrongly predicted as buried.

### Performance of the Method for Prediction of Active-Site, Buried and Exposed Non-Active Site Residues

**FIGURE 3:**
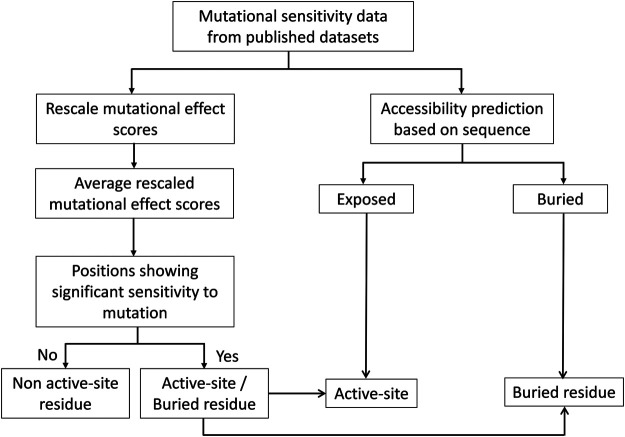
Flowchart of the methodology for prediction of active-site, buried and exposed non-active site residues. The mutational sensitivity score was determined for each mutant from deep sequencing-based screening. These scores were rescaled and averaged across each position. The sequence-based surface accessibility was predicted using the PROF server. The residues that showed significant sensitivity to mutations and which were predicted to be exposed were further considered to be the active-site residues.

Deep mutational scanning plays a crucial role in identifying protein-ligand interfaces and is useful regardless of the structural context. To identify the active-site residues and distinguish them from buried residues, we analyzed the structures of 11 proteins for the dataset used. The dataset comprises proteins that share interfaces with other proteins and includes a protein that binds to DNA. For all the proteins, structure-based solvent accessibilities were calculated to validate the predicted accessibilities (Figure 3).

For the prediction of active-site residues, an average sensitivity of ∼61% was observed (Table 3). This shows that if only the mutational sensitivity scores and sequence-based accessibility values are used, then those residues which are exposed and non-interacting, as well as ones that are buried, are segregated from the active-site residues. There is often a trade-off between specificity and sensitivity. Consistent with this, it was observed that for some of the datasets, there is low sensitivity, i.e., not all active-site residues are identified. In these cases, most of the exposed active-site residues have been incorrectly predicted as buried residues.

**TABLE 3:** Active-site prediction based on the mutational sensitivity data and PROF predicted sequence-based accessibility values.

Dataset	Sensitivity (%)	Specificity (%)	Accuracy (%)	Matthews correlation coefficient
Aminoglycoside kinase	72.7	87.7	87.1	0.34
BRCA1 RING domain-BARD1 binding	45.5	91.4	85.2	0.37
BRCA1 RING domain–E3 ligase activity	50	92.3	86.6	0.42
CcdB	75	98.9	96.9	0.79
Gal4 (DBD)	46.6	86.1	75.9	0.34
GB1 (IgG-binding domain)	85.7	91.5	90.7	0.66
Hsp90 (ATPase domain)	93.3	92	92.1	0.63
NUDT15	50	91.2	85.4	0.41
Pab1 (RRM domain)	62.5	87.9	85.1	0.41
PSD (pdz3 domain)	75	90.6	89.2	0.53
TEM1 β-lactamase	66.6	85.8	85.2	0.26
Ubiquitin	70	96.4	92.3	0.69

It has been observed that active-sites, as well as buried positions, have high mutational sensitivity. Therefore, it is essential that these buried positions are separated from the exposed active-site residues to enhance the accuracy of active-site prediction. To identify buried residues, we employed predicted accessibility values that have been obtained from sequence information. Since sequence-based accessibility Z-scores for buried residues are typically very low, these scores are subtracted from the averaged mutational sensitivity scores to predict them in the absence of structural information. After combining both averaged mutational sensitivity scores and sequence-based accessibility values from PROF, an average specificity of ∼90% is observed (Table 4). The sensitivity is ∼55% as some buried residues are predicted as exposed by the sequence-based accessibility predictor. The overall value of average sensitivity is affected by the low sensitivity of predictions in the case of HSP90 (Mishra et al., 2016). The pattern of mutational sensitivity for the buried positions in this protein is atypical, relative to the overall trend observed in the other large-scale mutagenesis datasets, with many buried positions tolerating charged substitutions. A similarly high degree of tolerance is observed for the BRCA1 RING domain, but only when BARD1 binding, rather than E3 ligase activity is assayed.

**TABLE 4:** Prediction of buried sites based on mutational sensitivity data and PROF predicted sequence-based accessibility values.

Dataset	Sensitivity (%)	Specificity (%)	Accuracy (%)	Matthews correlation coefficient
Aminoglycoside kinase	66.6	90.8	85.6	0.57
BRCA1 RING domain-BARD1 binding	38.5	88.2	80.2	0.27
BRCA1 RING domain–E3 ligase activity	38.5	80.6	73.3	0.12
CcdB	68.4	96.1	90.6	0.69
Gal4 (DBD)	50	78.6	77.6	0.13
GB1 (IgG-binding domain)	70	90.9	87	0.58
Hsp90 (ATPase domain)	18.8	84.7	73.5	0.06
NUDT15	69.7	88	84.2	0.55
Pab1 (RRM domain)	80	89.8	87.8	0.65
PSD (pdz3 domain)	55	90.5	81.9	0.48
TEM1 β- lactamase	59.8	91.5	80.9	0.55
Ubiquitin	45.5	83.3	76.9	0.26

The accuracy of prediction results for both active-site and buried residues are ∼88% and ∼84%, respectively. In the case of prediction of the buried positions, it was observed that the incorporation of sequence-based accessibility values played an important role in improving the results (Supplementary Figure S3). This helped to distinguish both the categories of mutationally sensitive positions, namely exposed active-site and buried positions. In contrast, prediction of the exposed non active-site residues prediction did not show significant improvement after incorporating the sequence-based accessibility scores (Supplementary Figure S3). Overall, incorporating the sequence-based accessibility values along with the averaged mutational sensitivity scores improves the prediction performance of the method primarily for buried residues and can be useful in identifying key residues in the protein even in the absence of structural information.

Along with the prediction of the active-site and buried residues, the exposed non active-sites can also be distinguished from the other two categories. The high value of sensitivity in these prediction results points to the ability of the method to identify these residues (Table 5). In a few cases, it was observed that there are a few exposed positions far from the active-site that show high mutational sensitivity. In the case of TEM1 β-lactamase, it was observed that exposed positions with large side chain show a high mutational sensitivity in comparison to the other exposed non active-site residues. For example, Trp210, Trp229 and Trp290 are exposed residues that are crucial for the structure and activity of β-lactamase (Huang et al., 1996). Mutations at such positions may lead to the instability of the enzyme, thus abrogating its function, though this needs to be confirmed by experiments. In comparison to predictions in the other two categories, prediction specificity was low for exposed non active-site residues, probably resulting from the lower fraction of true negatives in this category.

**TABLE 5:** Prediction of exposed non active-site residues based on mutational sensitivity data and PROF predicted sequence-based accessibility values.

Dataset	Sensitivity (%)	Specificity (%)	Accuracy (%)	Matthews correlation coefficient
Aminoglycoside kinase	94.9	22.1	76.1	0.25
BRCA1 RING domain-BARD1 binding	94.7	25	74.1	0.29
BRCA1 RING domain–E3 ligase activity	92.3	34.8	74.7	0.34
CcdB	97.1	29.6	78.1	0.39
Gal4 (DBD)	92.3	41.2	77.5	0.41
GB1 (IgG-binding domain)	89.2	35.3	72.2	0.29
Hsp90 (ATPase domain)	92.3	36.2	80	0.34
NUDT15	90.3	23.6	67	0.27
Pab1 (RRM domain)	94.2	50	81.1	0.52
PSD (pdz3 domain)	92.7	35.7	73.5	0.36
TEM1 β-lactamase	92.0	31	68.8	0.3
Ubiquitin	97.8	40	80	0.5

**FIGURE 4:**
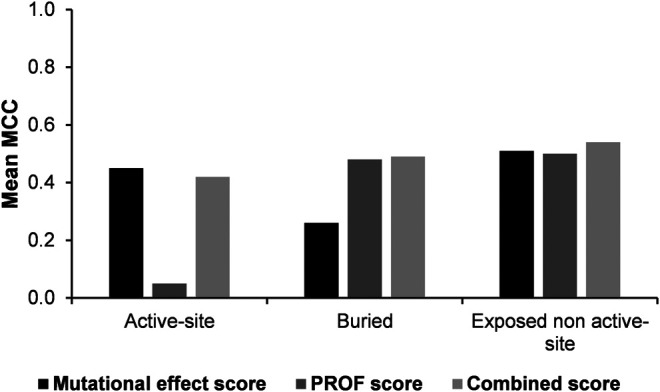
Comparison of Matthew’s correlation coefficients of predictions using experimental mutational effect scores alone, PROF predicted accessibility alone and experimental mutational effect scores combined with the PROF predicted accessibility scores (combined score).

The Matthew’s correlation coefficient (MCC) was computed using either the experimental mutational effect scores or the PROF predicted accessibility values. These values were compared with corresponding values obtained using the combined score for all the three categories of predictions (Figure 4). The results show that overall, the combined score yields the best results.

### Comparison of the Results Across Other Solvent Accessibility Predictors

In the above analysis, the sequence-based accessibility scores from PROF were considered along with the experimental mutagenesis scores to calculate the prediction sensitivity for the active-site, buried and exposed non active-site residues. The average Pearson’s correlation coefficient of predicted accessibility from PROF with calculated surface accessibility from NACCESS is 0.66.

**FIGURE 5:**
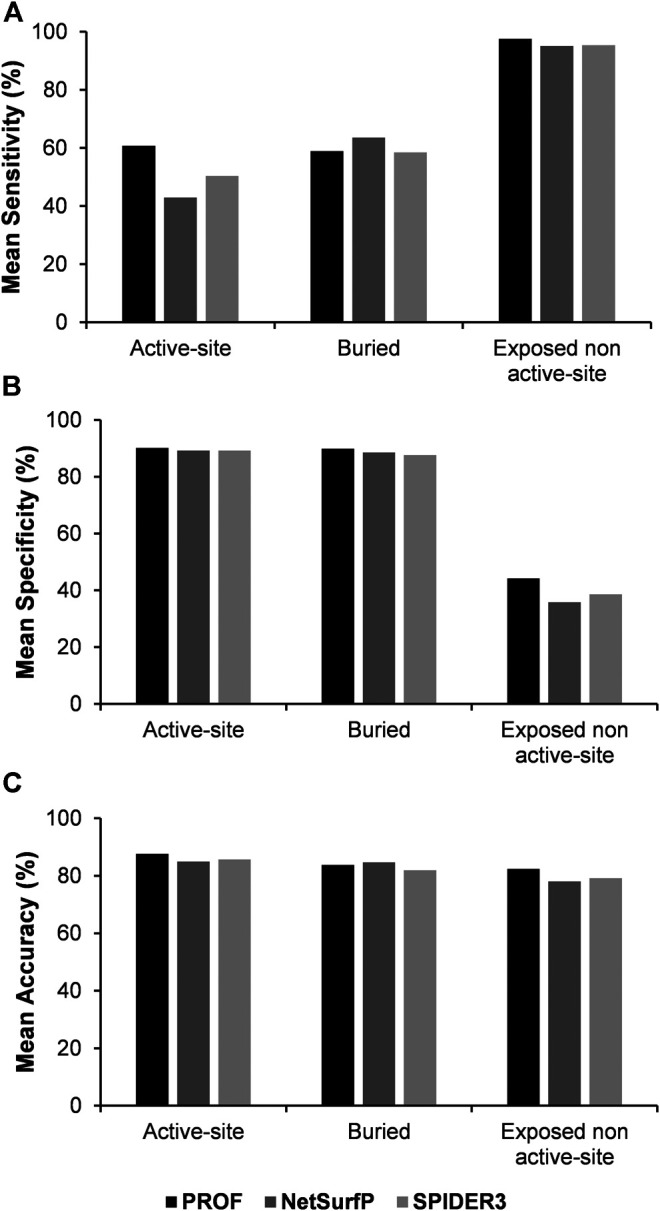
Comparison of mean values of sensitivity, specificity and accuracy of predictions using mutational effect scores combined with the predicted accessibility results from PROF, NetSurfP and SPIDER3 respectively.

The analysis was also performed with another sequence-based accessibility predictor NetSurfP (Petersen et al., 2009). In this case, the correlation between the predicted accessibility from NetSurfP and calculated accessibility from NACCESS, is improved with an average correlation coefficient of 0.72. The sensitivity, specificity and accuracy of the results were recalculated using NetSurfP rather than PROF for residue accessibility prediction (Figure 5, Supplementary Tables S3–S5). Results for prediction of buried and exposed non-active site residues are comparable with both accessibility predictors. However for active-site residues the sensitivity was lower when the NetSurfP predicted accessibility was used instead of the PROF predicted accessibility.

In order to compare between the various surface accessibility predictors, SPIDER3 (Heffernan et al., 2017) was also used for prediction of active-site, buried and exposed non active-site residues. SPIDER3 is a method that captures long-range, non-local interactions and predicts the protein one-dimensional structural properties. The correlation between the predicted accessibility using SPIDER3 and calculated accessibility using NACCESS was 0.68 which is comparable to the correlation coefficient observed in the case of PROF. After incorporating sequence-based accessibility scores from SPIDER3 with the experimental mutational sensitivity scores, there was a very slight improvement in the prediction sensitivity of the buried positions. However as with NetSurfP, the mean sensitivity values for prediction of active-site residues and mean accuracy values were lower with SPIDER3, relative to PROF (Figure 5; Supplementary Tables S3–S5).

### Comparison of the Results with Mutational Effect Predictors

**FIGURE 6:**
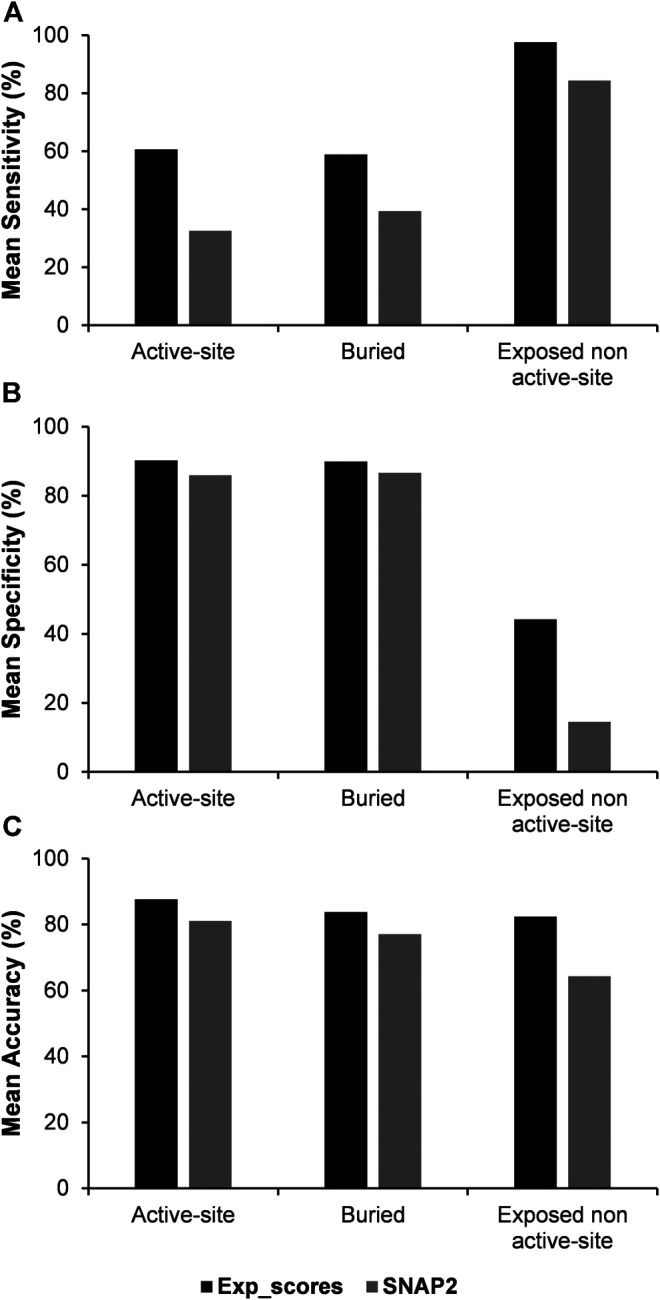
Comparison of mean values of sensitivity, specificity and accuracy of predictions using experimental and SNAP2 predicted mutational effect scores combined with the predicted accessibility results from PROF.

As there is currently a limited number of complete deep mutational scanning datasets, a similar analysis was carried out by using the predicted mutational effect scores from the computational variant effect predictor SNAP2 (Hecht et al., 2016), which required only the sequence as the input to predict mutational effect scores. An average Pearson’s correlation coefficient of 0.5 was see between the experimental and SNAP2 predicted scores. The three categories of residues namely, active-site, buried and exposed non active-site residues were further predicted using the SNAP2 scores by combining them with PROF predicted accessibility (Figure 6, Supplementary Table S6). The predicted variant effect scores poorly predict active-site residues. However, prediction metrics for buried and exposed non active-site residues are comparable in terms of their sensitivity, specificity and accuracy to those obtained with experimental mutational scores.

## Discussion

Deep mutational scanning is a method that is widely used to probe the effects of substitutions on proteins, which helps to identify functionally important residues (Adkar et al., 2012). In this study, we examined if such large-scale mutagenesis datasets, could be used to infer locations of functional sites in proteins and distinguish them from other positions based on their specific mutational sensitivity pattern.

The present analysis reveals that active-site residues are on average more sensitive to mutation than buried residues. Use of sequence-based accessibility predictions further contributes to distinguishing buried positions from the exposed active-site residues. The third category of residues that is largely insensitive to mutation, is exposed non active-site residues There are a few exposed non active-site residues that are mis predicted as active-site residues. One of the reasons for this is their proximity to the active-sites, thus making them sensitive to substitutions. In some cases, these exposed mutationally sensitive residues have accessibility values that are close to the cut-off that is used for classifying them as exposed or buried. Among the datasets considered for prediction of active-site residues, there is one deep mutational scan of the DNA-binding domain (DBD) of Gal4, a yeast transcription factor (Kitzman et al., 2015). Gal4 binds DNA as a homodimer via a Zn_2_Cys_6_-class domain centered on a pair of Zn^2+^ ions. This helps to maintain the fold of the DNA-binding residues. Substitutions at any of six cysteines completely disrupts the function (Marmorstein et al., 1992). Since these cysteines are both buried, but also involved in the activity of the protein, they are considered as active-site residues for analysis. They have been further excluded in the prediction of the buried residues. It was also observed that sensitivity of predictions decreases for proteins with a large number of interacting partners or with limited mutational sensitivity data. Thus, for the present study only those deep mutational scanning datasets are considered which have an average of at least ten mutants per residue.

For datasets where the relative fitness effects of single amino acid mutations were observed under antibiotic selection, an optimum antibiotic concentration value was selected for prediction. In the case of TEM1 β-lactamase, mutational data foe selection with an ampicillin concentration of 625 μg/ml were used (Stiffler et al., 2015). Higher concentrations of ampicillin result in high mutational sensitivity across the entire protein. This results in inability to separate the key catalytic residues from the non-interacting ones. For aminoglycoside kinase, the relative abundance of mutant vs. wild-type amino acids at each position was examined under kanamycin selection at a range of inhibitory concentrations (Melnikov et al., 2014). At high kanamycin concentration, the mutational sensitivity was again very high, thus data from the lower kanamycin concentration was used for analyzing the pattern of mutational sensitivity. In general, it appears that mutational scanning datasets are most useful when phenotypic screens are carried out under conditions where ∼25% of substitutions yield measurable phenotypes.

Amongst the deep mutational scanning datasets analyzed in this study, there are a few cases where there is high mutational sensitivity at non active-site residues. One such example is the deep mutational scan of TEM1 β-lactamase (Stiffler et al., 2015). There are residues that are distal from the active-site but are highly sensitive to substitutions, suggesting possible allostery (Avci et al., 2016). However, it is difficult to know if such mutational sensitivity is because of functional allostery or because of a decreased level of secreted protein, for example because of increased proteolysis. This emphasizes the need to measure both levels of properly folded protein as well as activity. This is not done in most mutational scans.

Since there still relatively few proteins that have been subjected to deep mutational scans, computationally predicted variant effect scores were used in place of experimental data. However, this led to poor predictions for active-site residues. In future, given recent advances in deep learning based structure prediction (Senior et al., 2020), it would be interesting to map computationally predicted variant scores onto structural models to more accurately predict active-site residues.

In addition to identifying buried, active-site and exposed non active-site residues, the present analysis has identified puzzling mutational sensitivity features in some of the proteins in the present dataset, that reflect either our incomplete understanding of determinants of protein stability and function or potential lacunae in the experimental data that need additional validation through repeat experiments.

## Data Availability

The original contributions presented in the study are included in the article/Supplementary Material, further inquiries can be directed to the corresponding author.

## References

[B1] Adkar B. V. Tripathi A. Sahoo A. Bajaj K. Goswami D. Chakrabarti P. (2012). Protein model discrimination using mutational sensitivity derived from deep sequencing. Structure 20, 371–381. 10.1016/j.str.2011.11.021 22325784

[B2] Aloy P. Querol E. Aviles F. X. Sternberg M. J. E. (2001). Automated structure-based prediction of functional sites in proteins: Applications to assessing the validity of inheriting protein function from homology in genome annotation and to protein docking. J. Mol. Biol. 311, 395–408. 10.1006/jmbi.2001.4870 11478868

[B3] Avci F. G. Altinisik F. E. Vardar Ulu D. Ozkirimli Olmez E. Sariyar Akbulut B. (2016). An evolutionarily conserved allosteric site modulates beta-lactamase activity. J. Enzyme Inhib. Med. Chem. 31, 33–40. 10.1080/14756366.2016.1201813 27353461

[B4] Bajaj K. Dewan P. C. Chakrabarti P. Goswami D. Barua B. Baliga C. (2008). Structural correlates of the temperature sensitive phenotype derived from saturation mutagenesis studies of CcdB. Biochemistry 47, 12964–12973. 10.1021/bi8014345 19006334

[B5] Berezin C. Glaser F. Rosenberg J. Paz I. Pupko T. Fariselli P. (2004). ConSeq: the identification of functionally and structurally important residues in protein sequences. Bioinformatics 20, 1322–1324. 10.1093/bioinformatics/bth070 14871869

[B6] Bloom J. D. Silberg J. J. Wilke C. O. Drummond D. A. Adami C. Arnold F. H. (2005). Thermodynamic prediction of protein neutrality. Proc. Natl. Acad. Sci. U.S.A. 102, 606–611. 10.1073/pnas.0406744102 15644440 PMC545518

[B7] Brzovic P. S. Rajagopal P. Hoyt D. W. King M.-C. Klevit R. E. (2001). Structure of a BRCA1– BARD1 heterodimeric RING–RING complex. Nat. Struct. Biol. 8, 833–837. 10.1038/nsb1001-833 11573085

[B8] Capra J. A. Laskowski R. A. Thornton J. M. Singh M. Funkhouser T. A. (2009). Predicting protein ligand binding sites by combining evolutionary sequence conservation and 3D structure. Plos Comput. Biol. 5, e1000585. 10.1371/journal.pcbi.1000585 19997483 PMC2777313

[B9] Chakravarty S. Varadarajan R. (1999). Residue depth: A novel parameter for the analysis of protein structure and stability. Structure 7, 723–732. 10.1016/s0969-2126(99)80097-5 10425675

[B10] Fischer J. D. Mayer C. E. Söding J. (2008). Prediction of protein functional residues from sequence by probability density estimation. Bioinformatics 24, 613–620. 10.1093/bioinformatics/btm626 18174181

[B11] Fowler D. M. Araya C. L. Fleishman S. J. Kellogg E. H. Stephany J. J. Baker D. (2010). High-resolution mapping of protein sequence-function relationships. Nat. Methods 7, 741–746. 10.1038/nmeth.1492 20711194 PMC2938879

[B12] Fowler D. M. Fields S. (2014). Deep mutational scanning: A new style of protein science. Nat. Methods 11, 801–807. 10.1038/nmeth.3027 25075907 PMC4410700

[B13] George R. A. Spriggs R. V. Bartlett G. J. Gutteridge A. MacArthur M. W. Porter C. T. (2005). Effective function annotation through catalytic residue conservation. Proc. Natl. Acad. Sci. U.S.A. 102, 12299–12304. 10.1073/pnas.0504833102 16037208 PMC1178014

[B14] Gherardini P. F. Helmer-Citterich M. (2008). Structure-based function prediction: approaches and applications. Brief. Funct. Genomic Proteomic 7, 291–302. 10.1093/bfgp/eln030 18599513

[B15] Gray V. E. Hause R. J. Fowler D. M. (2017). Analysis of large-scale mutagenesis data to assess the impact of single amino acid substitutions. Genetics 207, 53–61. 10.1534/genetics.117.300064 28751422 PMC5586385

[B16] Gupta K. Varadarajan R. (2018). Insights into protein structure, stability and function from saturation mutagenesis. Curr. Opin. Struct. Biol. 50, 117–125. 10.1016/j.sbi.2018.02.006 29505936 PMC6078801

[B17] Hecht M. Bromberg Y. Rost B. (2016). Better prediction of functional effects for sequence variants from VarI-SIG 2014: identification and annotation of genetic variants in the context of structure, function and disease. BMC Genomics 16, 1–12. 10.1186/1471-2164-16-S8-S1 PMC4480835 26110438

[B18] Heffernan R. Yang Y. Paliwal K. Zhou Y. (2017). Capturing non-local interactions by long short-term memory bidirectional recurrent neural networks for improving prediction of protein secondary structure, backbone angles, contact numbers and solvent accessibility. Bioinformatics 33, 2842–2849. 10.1093/bioinformatics/btx218 28430949

[B19] Huang W. Petrosino J. Hirsch M. Shenkin P. S. Palzkill T. (1996). Amino acid sequence determinants of beta-lactamase structure and activity. J. Mol. Biol. 258, 688–703. 10.1006/jmbi.1996.0279 8637002

[B20] Hubbard S. J. Thornton J. M. (1993). “NACCESS” computer program. London, United States: Department of Biochemistry and Molecular Biology, University College London.

[B21] Kitzman J. O. Starita L. M. Lo R. S. Fields S. Shendure J. (2015). Massively parallel single-amino-acid mutagenesis. Nat. Methods 12, 203–206. 10.1038/nmeth.3223 25559584 PMC4344410

[B22] Lichtarge O. Bourne H. R. Cohen F. E. (1996). An evolutionary trace method defines binding surfaces common to protein families. J. Mol. Biol. 257, 342–358. 10.1006/jmbi.1996.0167 8609628

[B51] Loris R. Dao-Thi M. H. Bahassi E. M. Van Melderen L. Poortmans F. Liddington R. (1999). Crystal structure of CcdB, a topoisomerase poison from E. coli. J. Mol. Biol. 285 (4), 1667–1677. 10.1006/jmbi.1998.2395 9917404

[B23] Marmorstein R. Carey M. Ptashne M. Harrison S. C. (1992). DNA recognition by GAL4: structure of a protein-DNA complex. Nature 356, 408–414. 10.1038/356408a0 1557122

[B24] McLaughlin R. N. Poelwijk F. J. Raman A. Gosal W. S. Ranganathan R. (2012). The spatial architecture of protein function and adaptation. Nature 491, 138–142. 10.1038/nature11500 23041932 PMC3991786

[B25] Melamed D. Young D. L. Gamble C. E. Miller C. R. Fields S. (2013). Deep mutational scanning of an RRM domain of the Saccharomyces cerevisiae poly(A)-binding protein. RNA 19, 1537–1551. 10.1261/rna.040709.113 24064791 PMC3851721

[B26] Melnikov A. Rogov P. Wang L. Gnirke A. Mikkelsen T. S. (2014). Comprehensive mutational scanning of a kinase *in vivo* reveals substrate-dependent fitness landscapes. Nucleic Acids Res. 42, e112–e118. 10.1093/nar/gku511 24914046 PMC4132701

[B27] Mishra P. Flynn J. M. Starr T. N. Bolon D. N. A. (2016). Systematic mutant analyses elucidate general and client-specific aspects of Hsp90 function. Cell Rep. 15, 588–598. 10.1016/j.celrep.2016.03.046 27068472 PMC4838542

[B28] Moretti R. Fleishman S. J. Agius R. Torchala M. Bates P. A. Kastritis P. L. (2013). Community-wide evaluation of methods for predicting the effect of mutations on protein-protein interactions. Proteins 81, 1980–1987. 10.1002/prot.24356 23843247 PMC4143140

[B29] Olson C. A. Wu N. C. Sun R. (2014). A comprehensive biophysical description of pairwise epistasis throughout an entire protein domain. Curr. Biol. 24, 2643–2651. 10.1016/j.cub.2014.09.072 25455030 PMC4254498

[B30] Petersen B. Petersen T. Andersen P. Nielsen M. Lundegaard C. (2009). A generic method for assignment of reliability scores applied to solvent accessibility predictions. BMC Struct. Biol. 9, 51. 10.1186/1472-6807-9-51 19646261 PMC2725087

[B31] Roscoe B. P. Thayer K. M. Zeldovich K. B. Fushman D. Bolon D. N. A. (2013). Analyses of the effects of all ubiquitin point mutants on yeast growth rate. J. Mol. Biol. 425, 1363–1377. 10.1016/j.jmb.2013.01.032 23376099 PMC3615125

[B32] Rost B. Sander C. (1994). Combining evolutionary information and neural networks to predict protein secondary structure. Proteins 19, 55–72. 10.1002/prot.340190108 8066087

[B33] Senior A. W. Evans R. Jumper J. Kirkpatrick J. Sifre L. Green T. (2020). Improved protein structure prediction using potentials from deep learning. Nature 577, 706–710. 10.1038/s41586-019-1923-7 31942072

[B34] Starita L. M. Young D. L. Islam M. Kitzman J. O. Gullingsrud J. Hause R. J. (2015). Massively parallel functional analysis of BRCA1 RING domain variants. Genetics 200, 413–422. 10.1534/genetics.115.175802 25823446 PMC4492368

[B35] Stiffler M. A. Hekstra D. R. Ranganathan R. (2015). Evolvability as a function of purifying selection in TEM-1 β-lactamase. Cell 160, 882–892. 10.1016/j.cell.2015.01.035 25723163

[B36] Strynadka N. C. Adachi H. Jensen S. E. Johns K. Sielecki A. Betzel C. (1992). Molecular structure of the acyl-enzyme intermediate in beta-lactam hydrolysis at 1.7 A resolution. Nature 359, 700–705. 10.1038/359700a0 1436034

[B37] Suiter C. C. Moriyama T. Matreyek K. A. Yang W. Scaletti E. R. Nishii R. (2020). Massively parallel variant characterization identifies NUDT15 alleles associated with thiopurine toxicity. Proc. Natl. Acad. Sci. U.S.A. 117, 5394–5401. 10.1073/pnas.1915680117 32094176 PMC7071893

[B38] Tan K. P. Varadarajan R. Madhusudhan M. S. (2011). DEPTH: A web server to compute depth and predict small-molecule binding cavities in proteins. Nucleic Acids Res. 39, W242–W248. 10.1093/nar/gkr356 21576233 PMC3125764

[B39] Tripathi A. Gupta K. Khare S. Jain P. C. Patel S. Kumar P. (2016). Molecular determinants of mutant phenotypes, inferred from saturation mutagenesis data. Mol. Biol. Evol. 33, 2960–2975. 10.1093/molbev/msw182 27563054 PMC5062330

[B40] Tripathi A. Varadarajan R. (2014). Residue specific contributions to stability and activity inferred from saturation mutagenesis and deep sequencing. Curr. Opin. Struct. Biol. 24, 63–71. 10.1016/j.sbi.2013.12.001 24721454

[B41] Wu N. C. Olson C. A. Du Y. Le S. Tran K. Remenyi R. (2015). Functional constraint profiling of a viral protein reveals discordance of evolutionary conservation and functionality. PLOS Genet. 11, e1005310–27. 10.1371/journal.pgen.1005310 26132554 PMC4489113

