# Supplementary material for: Prediction of Function Determining and Buried Residues Through Analysis of Saturation Mutagenesis Datasets
Source: Front Mol Biosci. 2021 Mar 11;8:635425. doi: 10.3389/fmolb.2021.635425 (PMC7991590; doi:10.3389/fmolb.2021.635425)
Supplement: Supplementary file 1 [file image1.pdf]

Supplementary Figures

Figure S1. Mutational effect scores from deep mutational scans of 12 proteins. The reported mutational effects scores were rescaled. Stacked histograms of the reported mutational effect scores (**A**) and rescaled mutational effect scores (**B**) are shown.

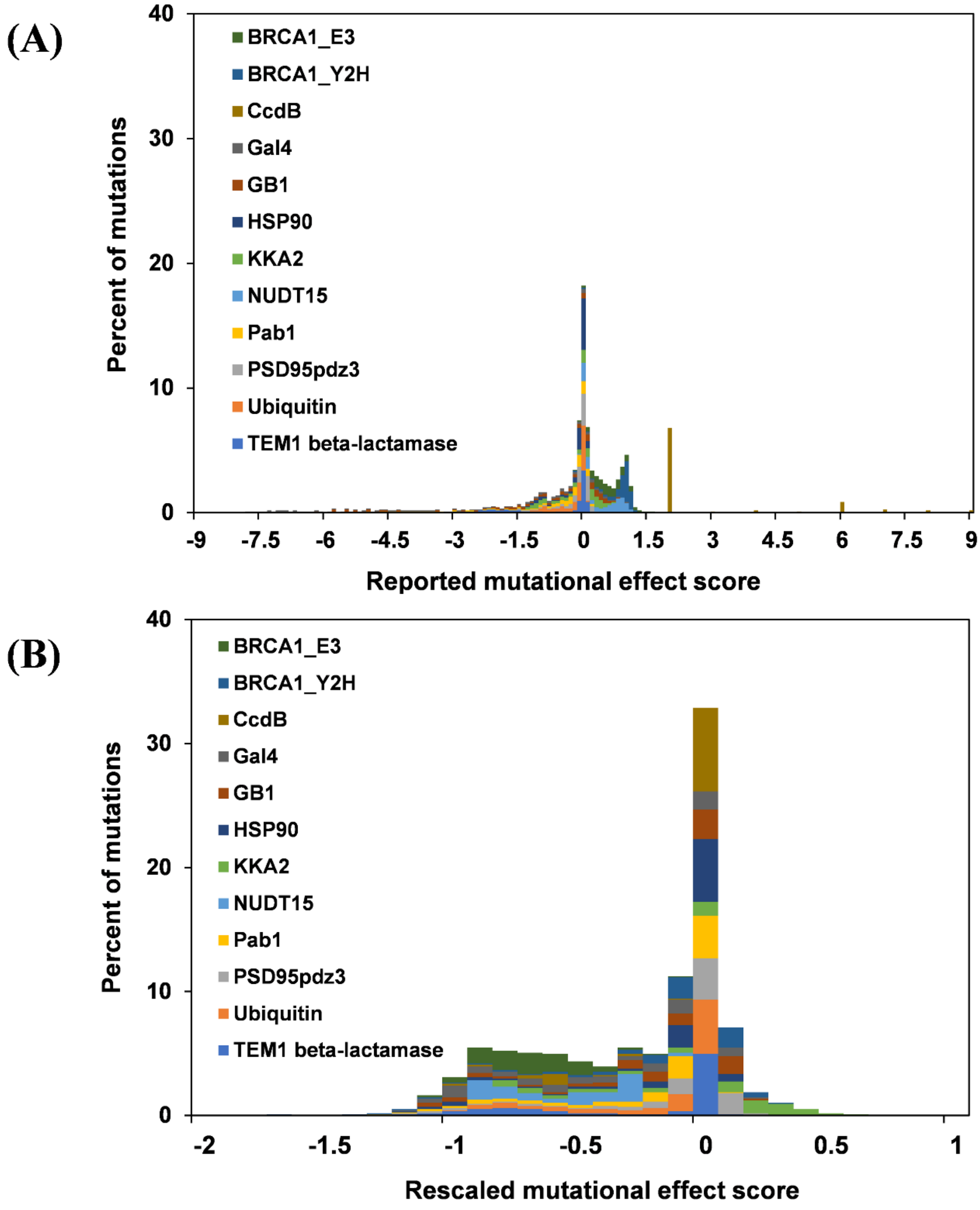

Figure S2. Mutational effects on protein activity inferred from phenotypic screening and deep sequencing. The rescaled mutational effect scores for active-site, buried (accessibility ≤ 5%) residues and exposed non active-site (accessibility > 5%) residues respectively. On the vertical axis, residues are grouped into (G, P), aliphatic (A–M), aromatic (F–W), polar (S–Q) and charged (D–R) amino acids. The horizontal and vertical axes denote the residue numbers and substitutions, respectively. Each heatmap is colored according to the rescaled mutational effect score of the mutant obtained from the published data. Green to red color gradation represents decreasing mutational effect scores and increasing mutational sensitivity. Mutational effect scores ≈ 0 for mutants that behave like wild type. Substitutions that were not sequenced in the input or selected pools or that were eliminated by subsequent quality filtration steps are shown in gray. The wild type residue at each position is indicated in white. Heatmaps for substitutions in the 12 large-scale mutagenesis datasets used are shown. (A) Aminoglycoside kinase (B) BRCA1 RING domain - BARD1 binding (C) BRCA1 RING domain ‑ E3 ligase activity (D) CcdB (E) Gal4 (DBD) (F) GB1 (IgG-binding domain) (G) Hsp90 (ATPase domain) (H) NUDT15 (I) Pab1(RRM domain) (J) PSD (pdz3 domain) (K) TEM1 β- lactamase (L) Ubiquitin.

(A) Aminoglycoside Kinase

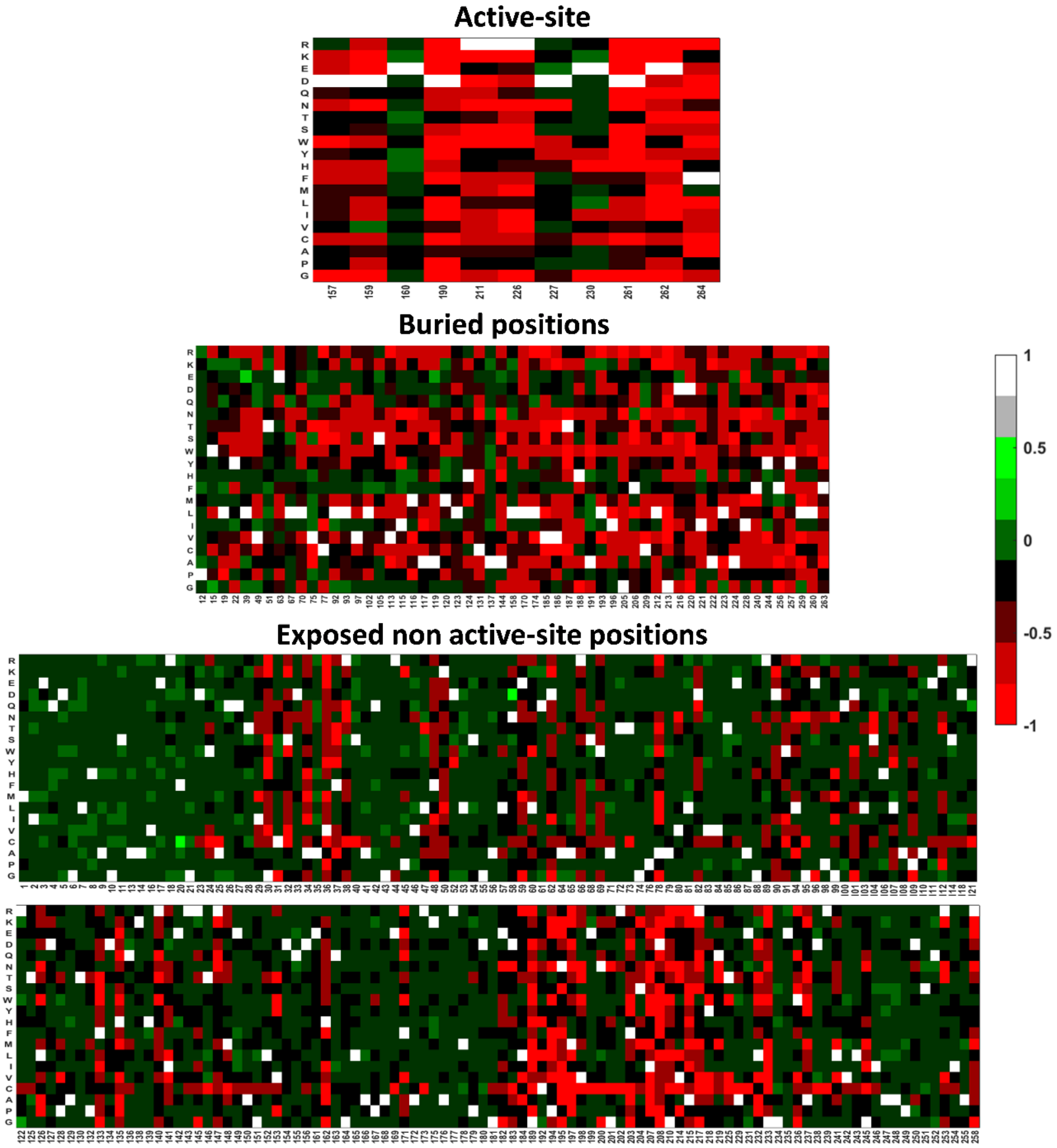

(B) BRCA1 RING domain - BARD1 binding

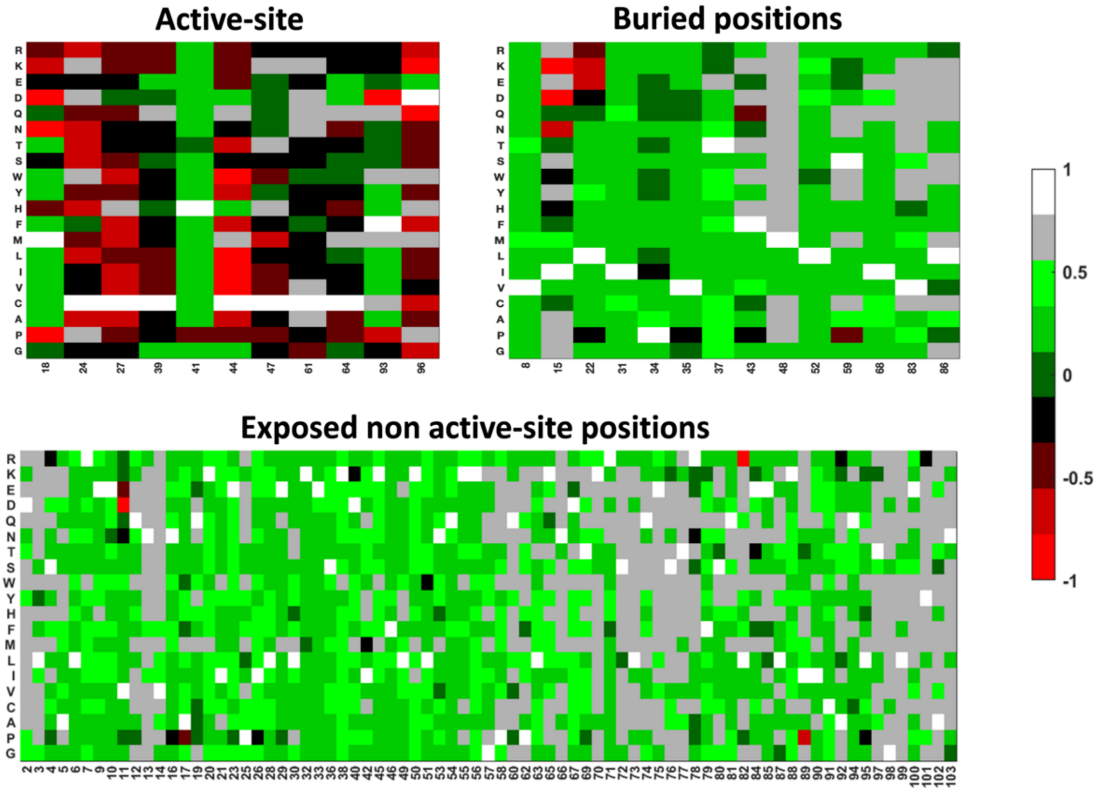

(C) BRCA1 RING domain – E3 ligase activity

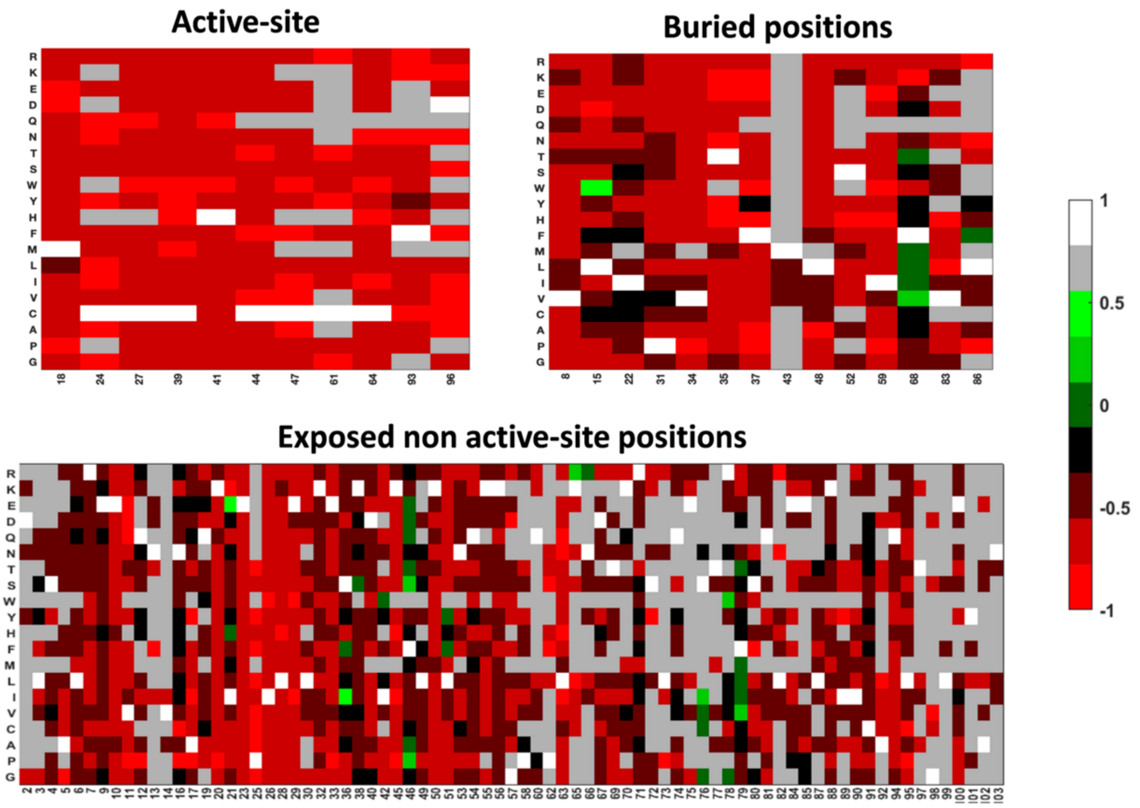

(D) CcdB

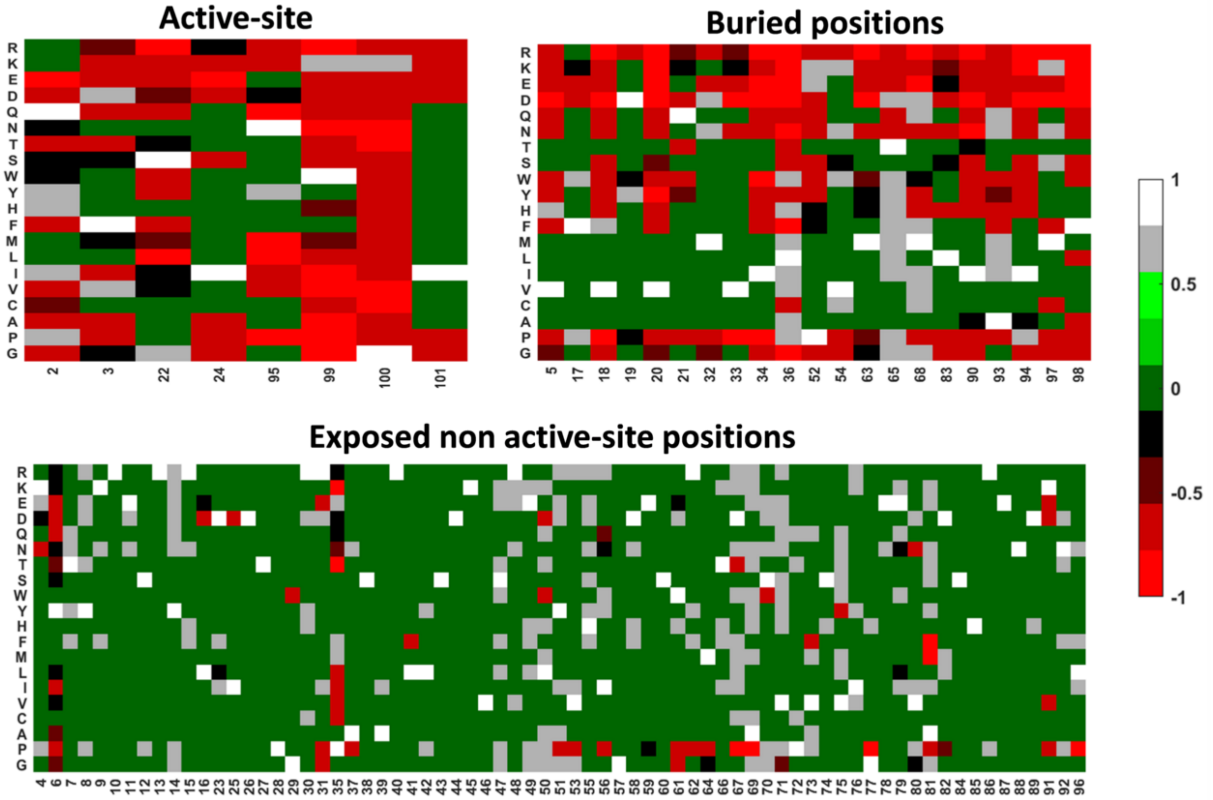

(E) Gal4 (DBD)

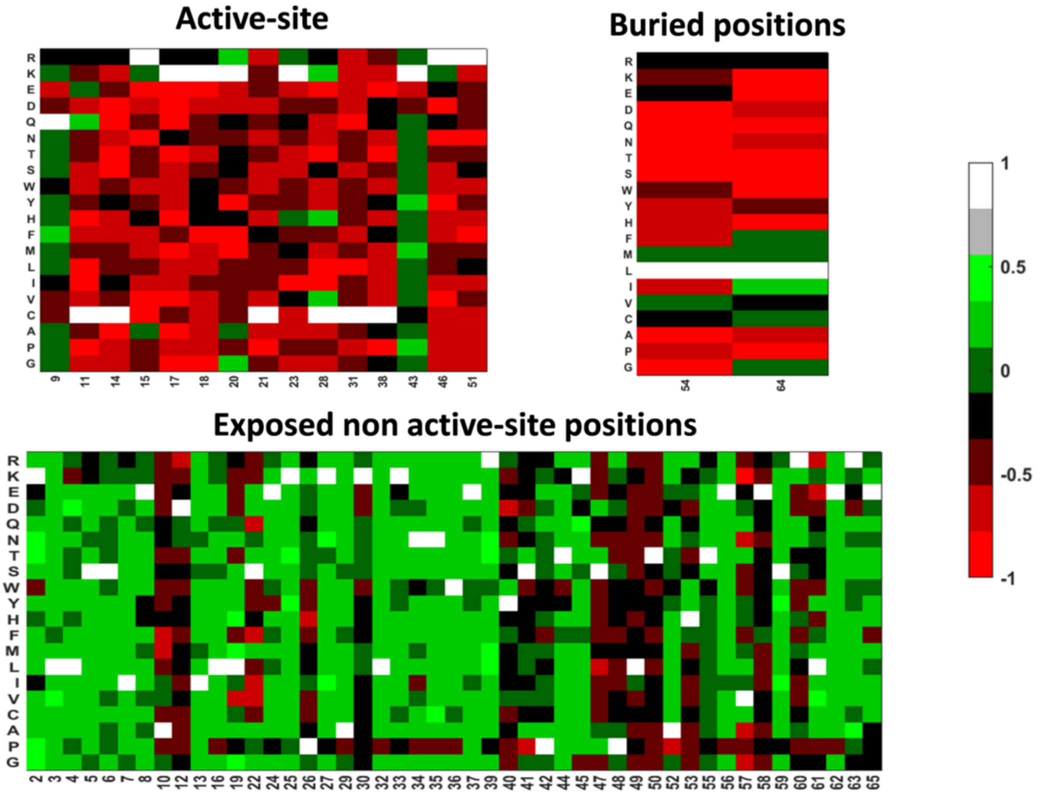

(F) GB1 (IgG-binding domain)

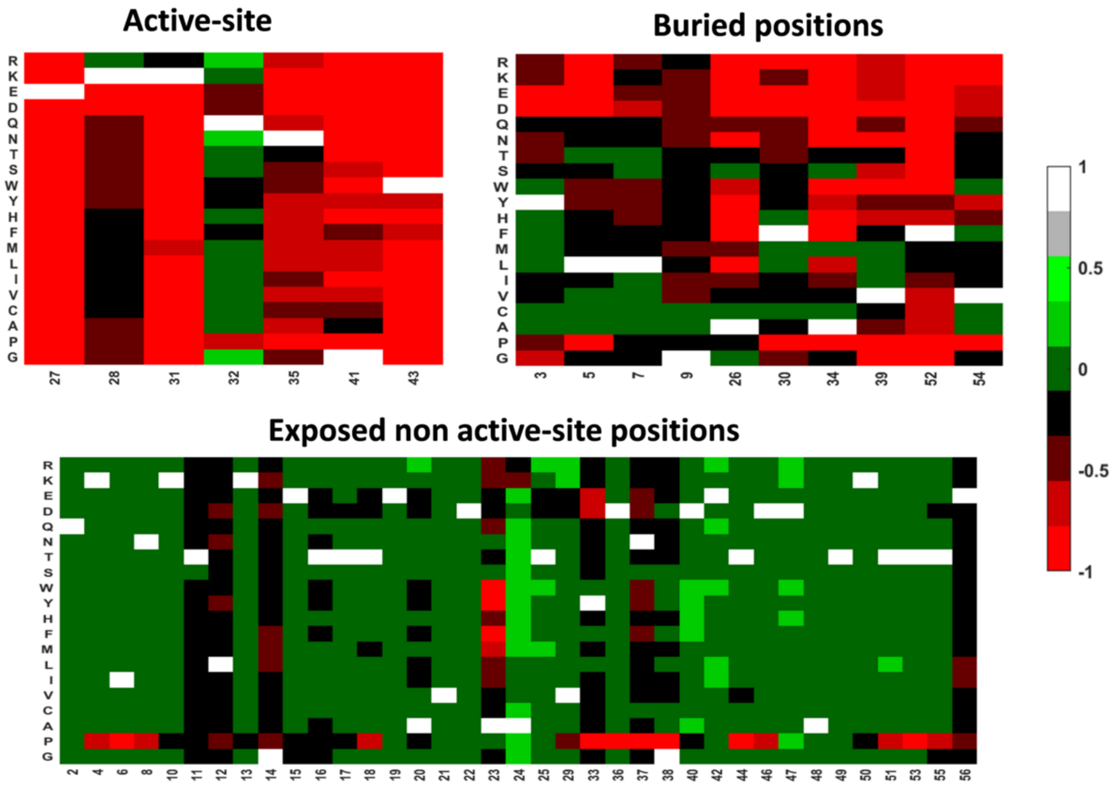

(G) Hsp90 (ATPase domain)

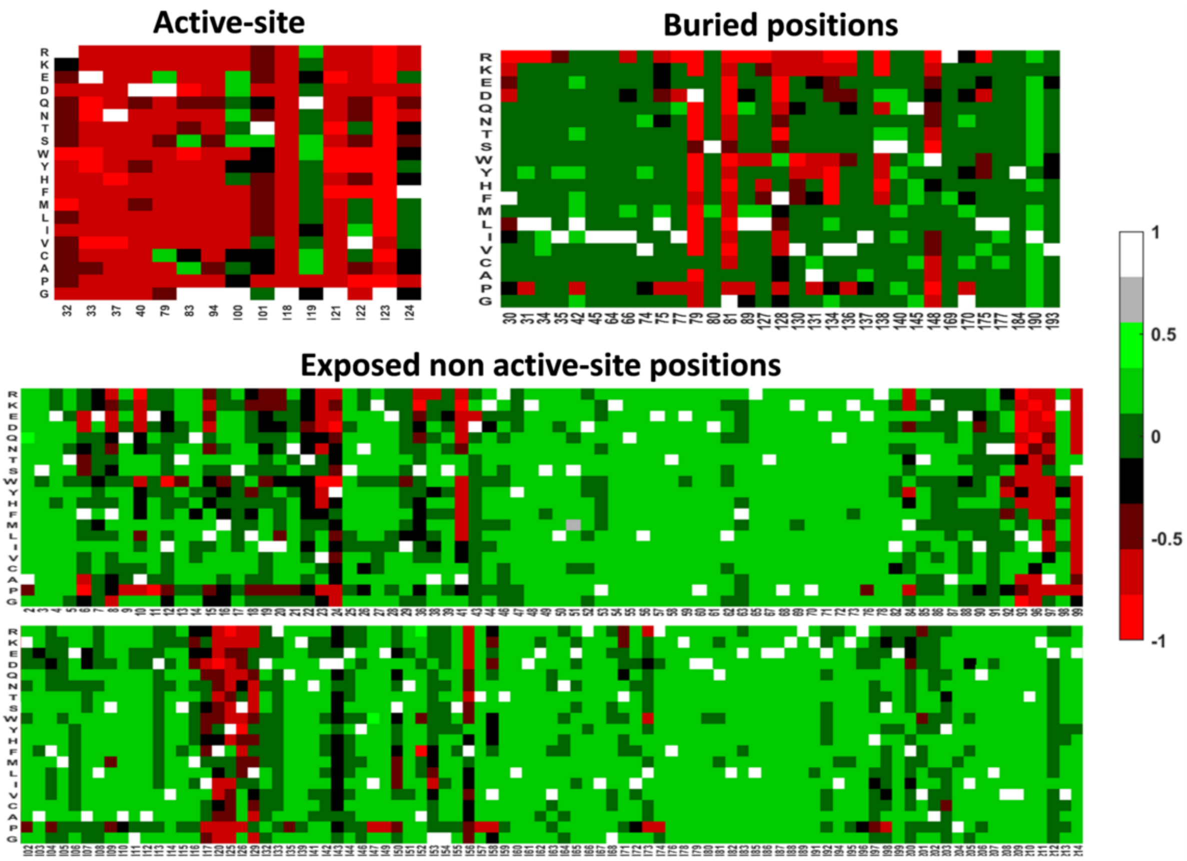

(H) NUDT15

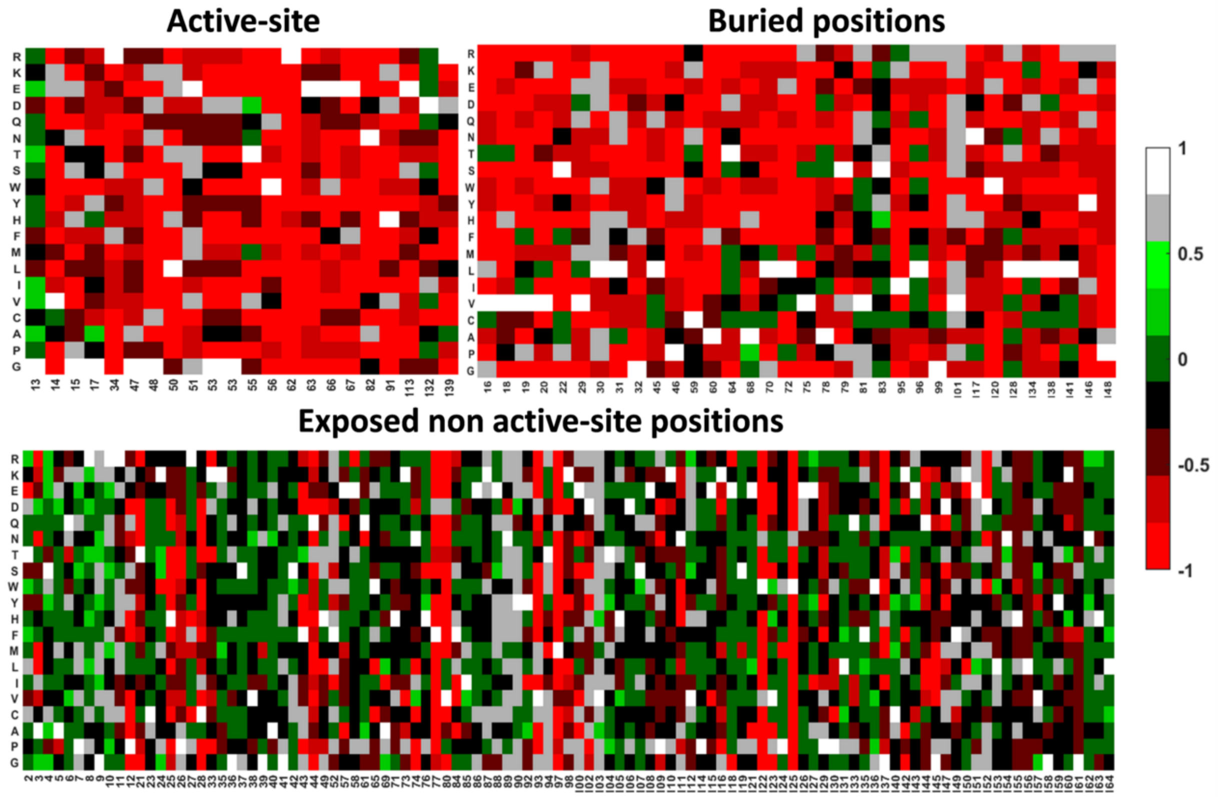

(I) Pab1(RRM domain)

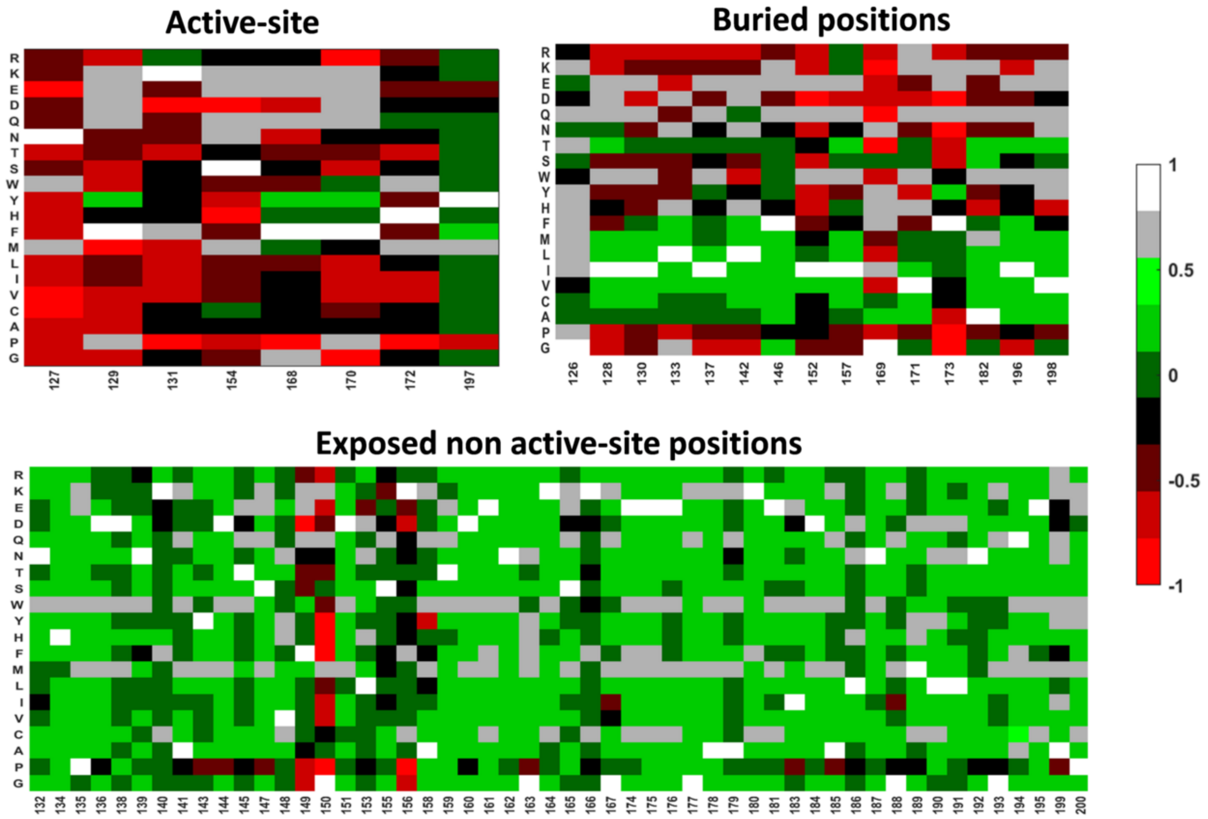

(J) PSD (pdz3 domain)

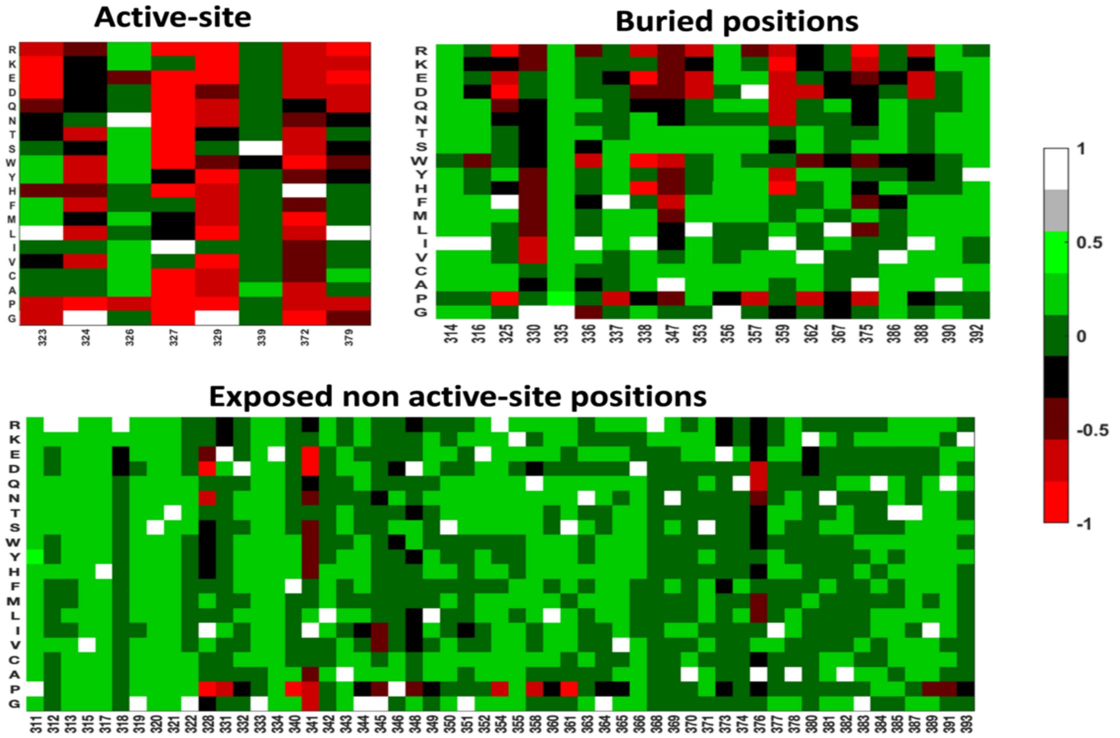

(K) TEM1 β- lactamase

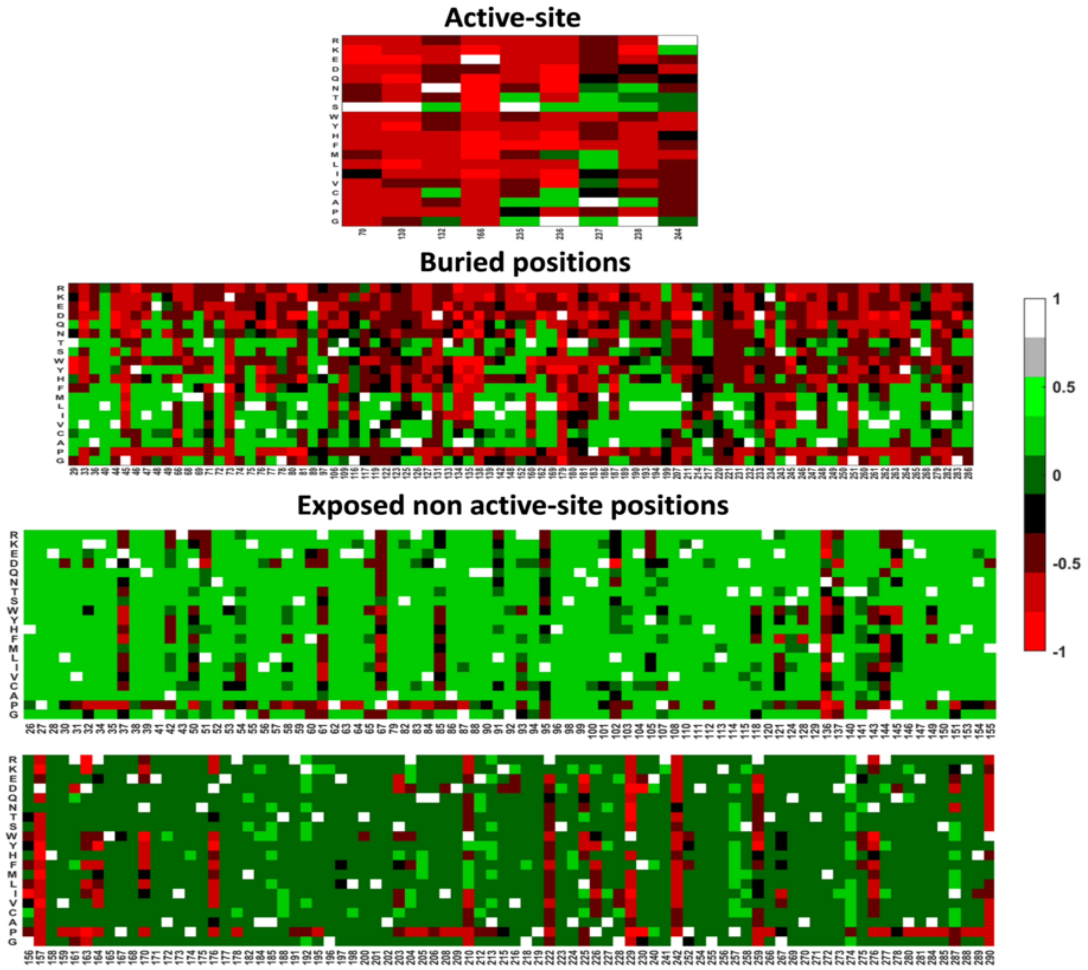

(L) Ubiquitin

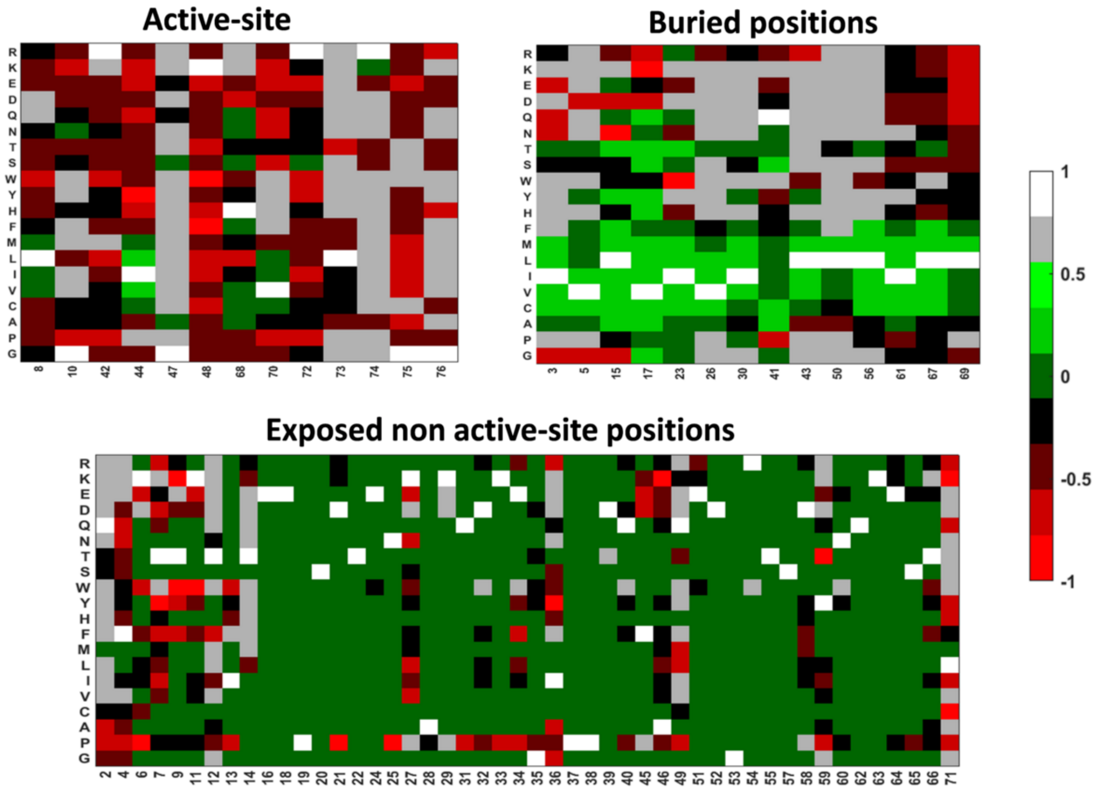
Active-site
Buried positions
Exposed non active-site positions
R K E D Q N T S W Y H F M L I V C A P G
8 10 42 44 47 48 68 70 72 73 74 75 76
3 5 15 17 23 26 30 41 43 50 56 61 67 69
1
0.5
0
-0.5
-1

Figure S3. Comparison of sensitivity of predictions using mutational effect scores alone and combined score (with inclusion of sequence-based accessibility values). The plots show the prediction of (A) active-site residues (B) buried residues (C) exposed non active-site residues.

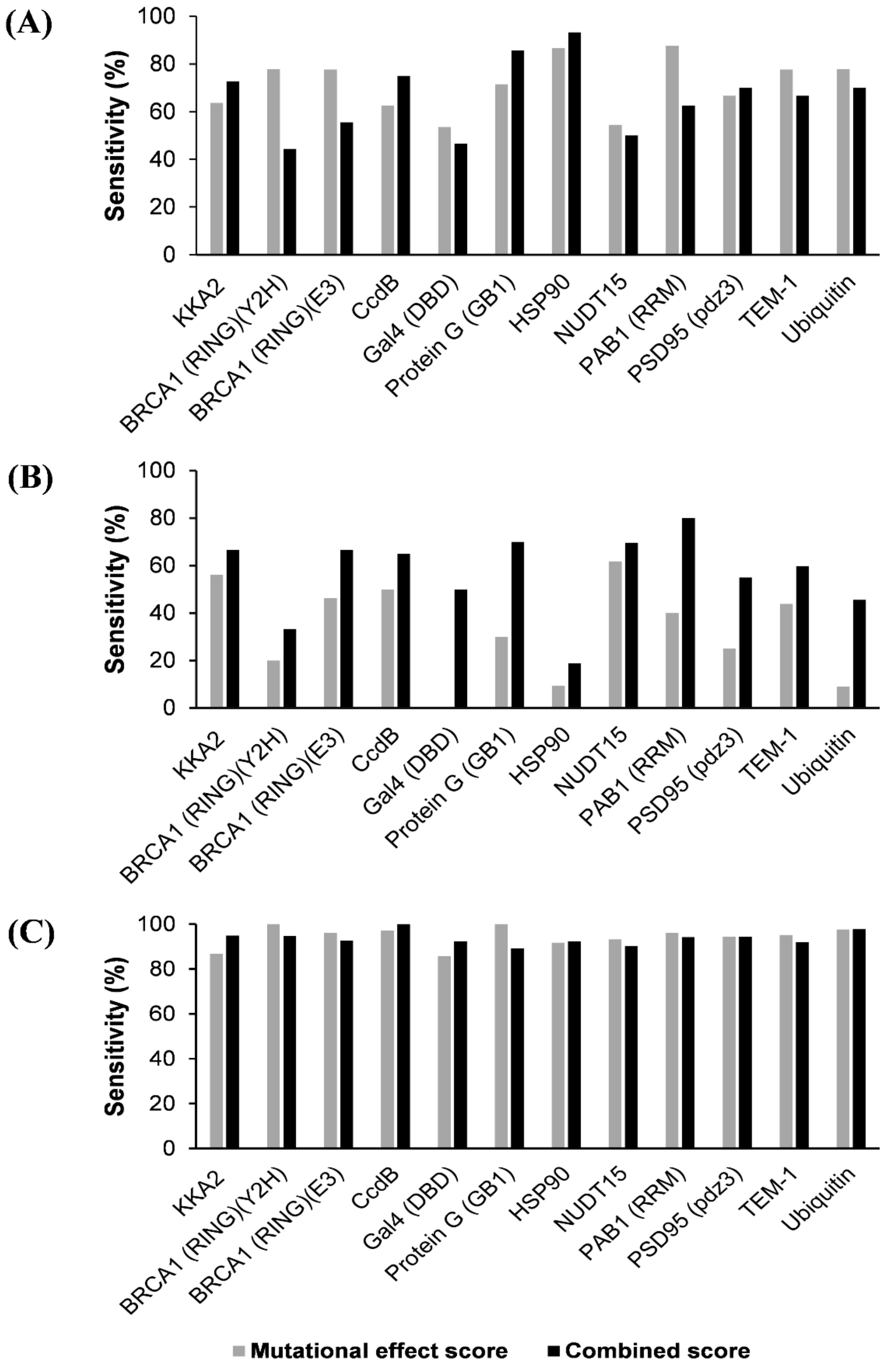

Supplementary Tables

Table S1. Original and rescaled mutational effect scores.

Table S2. Predicted and actual classification for buried, active-site and exposed non active-site residues. 'NA' indicates positions which have less than 10 mutants and hence were not subjected to classification.

Table S3. Active-site prediction based on the mutational sensitivity data and predicted sequence-based accessibility values using NetSurfP or SPIDER3.

Table S4. Prediction of buried sites based on mutational sensitivity data and predicted sequence-based accessibility values using NetSurfP or SPIDER3.

Table S5. Prediction of exposed non active-site residues based on mutational sensitivity data and predicted sequence-based accessibility values using NetSurfP or SPIDER3.

Table S6. Prediction of active-site, buried and exposed non active-site residues using SNAP2 predicted mutational effect scores combined with predicted accessibility results from PROF.
